# Bioinformatics resources for cancer research with an emphasis on gene function and structure prediction tools

**Published:** 2007-02-07

**Authors:** Daisuke Kihara, Yifeng David Yang, Troy Hawkins

**Affiliations:** 1 Department of Biological Sciences;; 2 Department of Computer Science;; 3 Markey Center for Structural Biology;; 4 The Bindley Bioscience Center, College of Science, Purdue University, West Lafayette, IN, 47907, USA

**Keywords:** microarray data management software, microarray data repository, gene function prediction, protein structure prediction, bioinformatics tools, protein-protein interaction database

## Abstract

The immensely popular fields of cancer research and bioinformatics overlap in many different areas, e.g. large data repositories that allow for users to analyze data from many experiments (data handling, databases), pattern mining, microarray data analysis, and interpretation of proteomics data. There are many newly available resources in these areas that may be unfamiliar to most cancer researchers wanting to incorporate bioinformatics tools and analyses into their work, and also to bioinformaticians looking for real data to develop and test algorithms. This review reveals the interdependence of cancer research and bioinformatics, and highlight the most appropriate and useful resources available to cancer researchers. These include not only public databases, but general and specific bioinformatics tools which can be useful to the cancer researcher. The primary foci are function and structure prediction tools of protein genes. The result is a useful reference to cancer researchers and bioinformaticians studying cancer alike.

## Introduction

Since its birth in the 1980s, bioinformatics has been rapidly growing, keeping pace with the expansion of genome sequence data. Recent technological development of large-scale gene expression analysis using DNA microarrays and proteomics experiments has further boosted the importance of bioinformatics methods. The integration of wet experiments and the use of bioinformatics analyses have become an indispensable part of the biological and clinical research of this century.

The area of cancer research is not an exception. A typical scenario of cancer research using bioinformatics tools is analysis of global profiles of gene expression in cancer (Hedenfalk et al 2002; Dressman et al 2003; Subramanian et al 2004; Glanzer and Eberwine 2004). Gene expression patterns of cancer cells are compared with those of normal cells or those of other subtypes of the cancer, and genes over/under-expressed in the cancer tissue are identified and clustered (identifying cancer signatures). Additional clinical questions include identifying signatures of metastasis (Weigelt et al 2005; Jones et al 2005) and prediction of clinical outcome (Chen et al 2005; Eschrich et al 2005). Then biological function of the genes of such signatures is also of biological and clinical interest, because they represent selected candidate genes for further biochemical investigation and for the development of targeted therapies, such as siRNA interference. Comparisons of findings across studies are very important.

Our review is organized to provide a sampling of the studies conducted to date, and to review the potential biological and clinical significance of the genes found in such signatures, hopefully to promote further follow-up development of novel routes to prevention and treatment. This review is organized as follows. First, we briefly list software for organizing microarray data and retrieving annotation information for genes from public databases. Next, we highlight several microarray data repositories. Then we review tools for function prediction of genes. In the subsequent section, protein structure prediction methods are reviewed. This is because a predicted tertiary and secondary structure can often give useful information for the design of biochemical experiments on a protein. Sometimes function of genes can be inferred from the predicted structure, too. Next, we review databases of protein-protein interaction. Information about interacting partners of a given gene can provide direct insight of the biochemical mechanism of a particular function of a cell and can also be a clue to guess about the function of that gene. This review is not intended to be a comprehensive survey of the field, but rather give a quick practical guide for recent developments of bioinformatics tools and databases useful for cancer research. Therefore in the choice of the introduced resources, preference is given to those that are non-commercial and well maintained. The bioinformatics tools and databases including those introduced in this article are available from our web site, http://dragon.bio.purdue.edu/bioinfolinks.

## Previous Reviews

Rhodes *et al* proposed a statistical model for performing meta-analysis of gene expression data across independent studies, and applied it to expression profiles of prostate cancer (Rhodes et al 2002). They identified the function of significantly differentially expressed genes by PubMed literature searches (Wheeler et al 2002) and a KEGG pathway query (Kanehisa et al 2004). In the study of expression profile analysis of colorectal cancer by Yeh *et al* functional characterization of up- and down-regulated genes was done using software to visualize expression patterns and function information of a set of genes was retrieved from public databases (Yeh et al 2005). Bono and Okazaki reviewed methods of function characterization of differently expressed genes using KEGG pathway mapping tools (Bono and Okazaki 2005). Statistical analysis of characteristic patterns of gene expression are practically very powerful in distinguishing cancer from normal tissue and distinguishing between subtypes of the cancer (Sorlie et al 2003). However, functional characterization of differently expressed genes can certainly give biological insight to the mechanism of the cancer. A recent excellent review by Rhodes and Chinnaiyan discusses the use of external functional information for interpreting and summarizing large cancer signatures (Rhodes and Chinnaiyan 2005). In their analysis, called the functional enrichment analysis, it is examined whether the difference of the fraction of genes which fall into a functional category from different samples is statistically significant or not.

In a functional analysis of a set of genes, it is desired that the employed method can assign accurate function to as large a number of genes as possible in the dataset. However, conventional homology search algorithms, such as BLAST (Altschul et al 1990) or FASTA (Pearson and Lipman 1988), can typically cover only 50% or less of the genes in a genome. Therefore it happens frequently that almost no functional clues are given to genes in a cluster of interest, which makes it extremely difficult to speculate about biological explanations to why the observed difference of gene expression profiles occurs. Note here that these homology search algorithms are also employed as a major computational procedure in public databases, such as KEGG and UniProt (Bairoch et al 2005), so that refereeing these databases does not necessarily solve the problem. One of the primary foci of this manuscript is to introduce and review bioinformatics tools for gene function and structure prediction, which aim to supplement functional assignment by the conventional homology search methods. Another focus is to introduce recent advanced protein structure prediction methods that will be useful for designing biochemical experiments of selected genes.

## Microarray Data Management and Analysis Software

Microarray studies of gene expression usually analyze hundreds to tens of thousands of genes. Typical questions to be asked involve the statistical significance of an observed differential expression pattern between samples, or the function of a set of genes with a different expression pattern. GoMiner, listed at the top of Table 1, is software designed to facilitate function analysis of a set of genes in microarray studies (Zeeberg et al 2003). Functions of a set of input genes are mapped onto the Gene Ontology (GO) tree, which is a hierarchically controlled vocabulary of gene function (Harris et al 2004). Function is assigned to genes by referring to public databases, such as UniProt, species specific databases at The Institute for Genome Research (TIGR) (Lee et al 2005), and Mouse Genome Informatics (MGI) (Eppig et al 2005). Up-regulated and down-regulated genes are flagged on the GO tree, and the relative enrichment of up-/down-regulated genes in a GO category is statistically tested. There are also links to other public databases including LocusLink (Pruitt and Maglott 2001), BioCarta (www.biocarta.com) and PDB (Berman et al 2000). Its recent upgraded version, named High-Throughput GoMiner, handles multiple microarray data, a feature which is useful for a time-course study of gene expression (Zeeberg et al 2005). GoSurfer has similar functionality to GoMiner, including visualization of gene function on the GO tree and statistical tests to search for the GO terms that are enriched in the annotations of a subset of input genes (Zhong et al 2004).

GenMAPP is designed to view and analyze microarray data on biological pathways (Dahlquist et al 2002; Doniger et al 2003). Input genes can be mapped onto a biological pathway, which can be one of the standard pathways imported from KEGG or a user-customized pathway. Up-regulated and down-regulated genes in an experiment can be shown in a different color on the pathway. From each box of genes in a pathway, a user can view function annotation in public databases including UniProt, MGI, and GO. The numerical values of the expression level can be also retrieved. MAPP Finder, an associated program to GenMAPP, can also employ the function enrichment analysis on the GO tree.

ArrayTrack is comprehensive microarray data management and analysis software (Tong et al 2004). Multiple microarray data can be stored in an organized fashion and standard statistical tests can be employed in order to detect genes with a significantly different expression pattern among samples. Data normalization methods available in this software facilitate cross-chip comparison. It also provides a collection of functional information about genes, proteins and pathways imported from public databases. The functional enrichment test on the GO tree can be performed, and also several data plotting and visualization tools are available.

We limited the list in Table 1 to include only software easily downloadable to a local machine and free for academic users. There is also free web-based software, including DAVID (Dennis, Jr. et al 2003) and Onto-Express (Draghici et al 2003).

The above software is mainly aimed to cluster genes based on function and for mapping pathways. Table 2 lists software for gene clustering using statistical methodologies. caGEDA provides many alternative statistical tools for each step in microarray data analysis (preprocessing, feature selection, and patient prediction model development) (Patel and Lyons-Weiler 2004). Users can easily perform comparative evaluation of different methods on their data sets.

Significance Analysis of Microarrays (SAM) (Tusher et al 2001) and NUDGE (Dean and Raftery 2005) use R, which is a language and environment for statistical computing and graphics (http://www.r-project.org/). The last website contains abundant links to statistical tools for gene expression analysis using R. A good summary of statistical testing for gene expression was given by Dudoit et al. (Dudoit et al 2003).

## Microarray Data Repositories

In this section, we briefly review public microarray repositories (Table 3). These repositories are very useful to retrieve data to perform cross-sample studies, identifying robust gene expression patterns across different conditions or different (sub)types of cancer (Rhodes and Chinnaiyan 2005). Data in the databases can also be analyzed using associated online tools. The Gene Expression Omnibus (GEO) at the National Center for Biotechnology Information (NCBI) holds the largest number of high-throughput gene expression data entries, which exceeds 54,000 at the time of writing of this manuscript (Barrett et al 2005). Data from non-array-based high-throughput experiments are also stored, including comparative genomic hybridization, serial analysis of gene expression (SAGE) and mass spectrometry peptide profiling. Individual “Sample” data are also organized into “Series”, which bring related Samples together with summary tables of the Series. Data mining and visualization tools, such as clustering methods, are available for most of the stored data. ArrayExpress is another public repository for microarray data hosted by the European Bioinformatics Institute (EBI) (Parkinson et al 2005). This is useful not only for retrieving data; expression patterns can be visualized by a collection of tools called Expression Profiler (Kapushesky et al 2004). This web-based tool kit includes tools for data preprocessing, clustering, visualization and comparison between multiple samples. CIBEX is another public database, together with GEO and ArrayExpress, recommended by the Microarray Gene Expression Data (MGED) society for storing expression data related to publications (Ikeo et al 2003). In addition to the three repositories, three additional large databases are listed in Table 3. SMD also provides database software developed originally for the authors’ own use. GXD is specific for the expression profiles of transcripts and proteins in different mouse strains and mutants (Hill et al 2004). Oncomine is specific for gene expression in cancer (Rhodes et al 2004).

## Protein Function Prediction Tools

Probably some of the most frequently used bioinformatics tools in cancer research are gene function prediction methods. As we have seen above, most of the microarray data management software import gene function from public databases, which typically hold function information of only up to half of the genes in a genome. In order to perform the functional enrichment analysis on microarray data, it is crucial that genes in a cluster of interest have annotated function. Here we introduce several interesting gene function prediction methods developed in recent years. These tools are aimed to give functional clue to genes beyond a conventional BLAST search. Function can be predicted from gene (amino acid) sequence, the tertiary structure, interacting partners, or of course, expression patterns of genes (Watson et al 2005). The focus of this section is sequence-based methods, because sequence information is usually available for all of the genes in a microarray analysis.

In Table 4, first, three homology search methods are listed. Although less distributed, FASTA performs better or at least comparable to BLAST (Brenner et al 1998). The site at Virginia University will provide also the local copy of the program. The database search results of course depend on the sequence database to be searched. If a recent version of the sequence database is not available at the Virginia site, it would be better to try the KEGG site at Kyoto University. PSI-BLAST (Altschul et al 1997) is a variant of BLAST. It performs an iterative search of a database using information of retrieved sequences from former rounds; hence generally it has better sensitivity than BLAST and FASTA. But at the same time, caution should be used in examining PSI-BLAST search output, because spurious hits can easily contaminate the results. We reemphasize here that function annotation in public databases is mainly derived by these homology search methods, thus running these methods in a standard fashion may not yield additional useful annotation. Therefore, these analyses may be performed when users want to try a different parameter set for a more aggressive search or a different database to be searched.

Pfam (Bateman et al 2002) is a database of protein families described by Hidden Markov models (HMM), which are statistical representations of multiple sequence alignments (Eddy 1996). Since a query sequence is searched against HMMs that have more information than single sequences, an increased sensitivity in the search is expected. From the Pfam website, a database search can be performed. Also the database itself and software for searching and creating a HMM database can be downloaded.

The next three resources, SMART (Letunic et al 2004), PROSITE (Hulo et al 2004) and ELM (Puntervoll et al 2003) are sequence motif databases with different features. SMART stores conserved regions in multiple sequence alignments of protein families, which can be used as signatures of each gene family. On the other hand, sequence motifs in PROSITE are primarily biologically significant sites described in literature, which include functional sites and sites which are subject to chemical modifications. ELM is a database for functional sites of eukaryotes.

STRING is an interactive database of known and predicted functional associations between genes (von Mering et al 2003). The interesting feature of STRING is that the function of a query sequence is predicted by comparative genomics methods, which are made possible by the growing number of complete genomes available. For example, if a query gene locates next to a gene of known function in several genomes of moderate evolutionary distance from each other, it would indicate that the query gene is involved in the same pathway or function as the adjacent gene. Genes that have the same phylogenetic profile (*i.e*. tree) and genes with the same pattern of co-occurrence and co-absence in genomes may also indicate that they are functionally linked. STRING also uses co-expression patterns in microarray analyses, and previous knowledge mined from PubMed literature abstracts. Users can perform function prediction on the web site, and also the functional association data in STRING are freely available.

PSORT is a server for predicting subcellular localization of genes (Nakai and Horton 1999). Basically, sequence features (signal sequences etc.) in a query sequence are detected and classified to known localization using a machine learning technique. The series of PSORT server families and links to the other servers of the same sort listed in the web site would be also useful.

The PFP (Protein Function Prediction) server was recently developed by our group (Hawkins and Kihara 2005a; Hawkins and Kihara 2005b). Unlike the conventional way to use PSI-BLAST, PFP mines more functional information from sequence hits with generally-thought insignificant hits by applying function association rules learned from genes of known function in public databases. PFP performed the best at the automatic function prediction competition held at the 13^th^ Annual International Conference on Intelligent Systems for Molecular Biology (ISMB) in June, 2005 (http://ffas.burnham.org/AFP).

Among the servers listed here, BLAST, FASTA and Pfam are the most reliable but may not provide additional functional information to annotation already stored in public databases. The other methods often outperform the three methods above and have a higher coverage, but should be used carefully because they also have a relatively high rate of spurious hits. A reasonable way to reduce false positives is to use different methods and compare the results to see if the prediction is consistent among the used methods.

## Protein Structure Prediction Tools

When candidates of genes are selected for experimental work-up by a microarray analysis, bioinformatics protein structure prediction tools are often very useful for designing biochemical experiments. For example, predicted secondary structure of a gene is a good clue to guess the domain structure of a gene, which is important to design limited proteolysis experiments in order to identify the functional region of the gene. The prediction accuracy of current secondary structure prediction algorithms is about 75% (Rost 2001; Kihara 2005), which would be high enough for routine use. Five secondary structure prediction tools are listed in Table 5. All of them use a machine learning technique to recognize known sequence patterns for α-helices and β-strands. PSI-PRED (Jones 1999), PORTER (Pollastri and McLysaght 2005), SABLE (Adamczak et al 2005) and PredictProtein (Rost and Sander 1994) use artificial neural networks, and SAM-T02 (Karplus et al 2003) uses the HMM. SABLE and PORTER claim the best accuracy in this field to date (78.4% and 79%, respectively). A local copy of the program is available for PSIPRED and SAM-T02. Although the accuracy of PredictProtein is relatively lower among those listed here, the server predicts not only the secondary structure but also other structural information, including disordered regions, coiled-coil regions, per residue solvent accessibility, and motifs in a query sequence. Thus it can be used as a convenient one-stop server for analyzing a protein sequence.

COILS predicts coiled-coil regions of a protein by recognizing unique patterns of periodic occurrence of hydrophobic residues in a sequence (Lupas 1996). Coiled-coil regions have been drawing attention recently because these regions are often binding sites to other proteins. GlobPlot (Linding et al 2003) and PONDR (Romero et al 2001) are prediction tools for intrinsic disordered regions of proteins, which do not have stable secondary structures in their native conformation.

Importance of disordered regions has also been recognized recently because many functionally important sites, *e.g*. those responsible for binding to other proteins or ligand molecules, are outside of the stable globular domains and thus intrinsically disordered. Programs for local use are available for all of three tools.

TMHMM (Sonnhammer et al 1998) and HMMTOP (Tusnady and Simon 2001) are transmembrane (TM) domain prediction tools which use HMM. TM domain prediction is one of the most successful structure predictions in bioinformatics (Kihara et al 1998). HMMTOP reports that 98% of the domains and 85% of topology of TM proteins in their benchmark set are correctly predicted. Both tools are web-based servers, and HMMTOP also provides a local copy of the program.

The bottom half of Table 5 lists protein tertiary structure prediction tools. Methodology of protein tertiary structure prediction has made dramatic improvements in the past decade, and the accuracy of some methods has reached a practical level. A recent review concisely describes the current status of this field (Schueler-Furman et al 2005). Structural prediction methods are roughly classified into three categories, namely homology modeling, threading (fold recognition), and “*ab initio*” or “*de novo*” folding (Jones 2000; Baker and Sali 2001; Forster 2002). Homology methods use an experimentally determined tertiary structure of a highly homologous protein to a query protein sequence as a template for modeling. Therefore, when an appropriate template structure is available in PDB, a very accurate model in an atomic detailed level can be built. SWISS-MODEL (Schwede et al 2003) and HHPred (Soding et al 2005) are web-based servers for homology modeling. The HHPred software is also available for download. MODELLER (Sali and Blundell 1993) is the most widely distributed and one of the earliest examples of this type of software. Both MODELLER and SWISS-MODEL have a database of homology models generated by the software.

The next three tools, FUGUE (Shi et al 2001), Phyre (Bates et al 2001) and SPARKS (Zhou and Zhou 2004) fall into the category of threading (Skolnick and Kihara 2001; Skolnick et al 2004). Threading algorithms seek a template protein in a database that structurally fits well to a query sequence. Unlike homology modeling, an apparent sequence similarity between a query sequence and a template protein is not a necessary condition. Threading methods have improved significantly in the past years, and can detect remotely related protein structures very well from a database, if any exist. A statistical score, the Z-score, shows the significance of the match between a query sequence and a template structure. Users should pay attention to the Z-score of retrieved models, and should only use models with a significant Z-score, as recommended by the server. When the Z-score is low, it may simply mean that there are no structures that fit well to a query, or the alignment between the query and the template is not very reliable.

The last server, Robetta (Kim et al 2004), is an *ab initio* method, which assembles a model from pieces of structural fragments retrieved from a database. Although algorithms of this category have also made a dramatic improvement (Kihara et al 2001; Skolnick et al 2003), it is still early to use *ab initio* methods routinely. When using *ab initio* methods, generated models should be checked carefully to see if they are reasonable in the biological sense based on background knowledge of the protein.

## Protein Protein Interaction Databases

The last group of resources we describe here are databases of protein-protein interactions (PPI) in model organisms (Table 6). In the past five years, an increasing number of large-scale experiments for revealing PPI in various organisms have been conducted, and most of the data are available at databases on the internet (Auerbach et al 2002). PPI of a gene is very important information to speculate the context of the gene’s role; for example, the pathway or subcellular localization of a gene. BIND (Alfarano et al 2005) is currently the largest PPI data repository, and contains over 200,000 interactions from more than 1,500 unique organisms. It also provides tools for visualization and data retrieval. DIP (Salwinski et al 2004) is one of the earliest databases of this kind and stores over 18,000 interactions. MIPS stores mammalian PPI data collected from literature with *Mus musculus* as the reference organism (Pagel et al 2005). HPRD is a unique database of information of human proteins in health and disease, including PPIs, post-translational modifications, disease associations, tissue expression etc., extracted manually from literature (Peri et al 2003). GRID stores PPI data of the fruit fry, yeast, and worm. Note that data is downloadable from all the databases above.

IntAct (Hermjakob et al 2004) and Ospray (Breitkreutz et al 2003) are an open source database and toolkit for storage, visualization and analysis of PPI data. These packages would be useful to integrate in a microarray data management system to link to PPI data.

## Summary

In the last decade, many new techniques have appeared in experimental biology that have had a tremendous impact on directions and styles of cancer research. And the same thing is true for bioinformatics databases and tools; indeed development and improvement of bioinformatics resources might be even more rapid than experimental techniques. A key to effectively handling large-scale experimental data is to use appropriate and reliable bioinformatics tools to organize and analyze that data.

**Figure 1.**
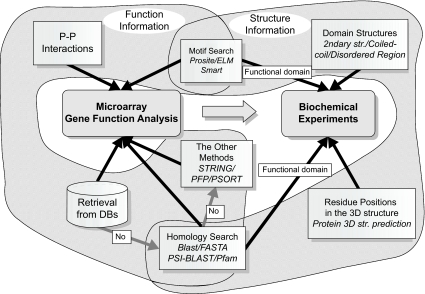
Bioinformatics tools for protein function and structure prediction. Gene function assignment is used in a microarray analysis. Structure information such as the domain structure or the tertiary structure of a certain gene is useful for designing biochemical experiments of the gene. See the text for the details.

The bioinformatics tools reviewed here were chosen with a scenario that gene-expression patterns of a certain type of cancer are investigated, functional enrichment analyses are performed to identify the signature of the cancer type, and further biochemical experiments are designed for a handful of selected genes with help of protein structure prediction methods (Fig. 1). If the function of genes cannot be retrieved from public databases, homology search methods are the first choice for prediction. If there are still no significant hits in the search, the other sequence based methods, including STRING, PFP, and PSORT can be used. At the same time, motif searches may also be able to provide functional clues for the genes. PPI data will provide the context of the genes’ function, and can be used to cluster genes in terms of their interaction patterns. To design biochemical experiments to determine functional/interaction domains of a given gene, it is helpful to predict the secondary structure of the gene. Motif search and homology search methods can also provide conserved functional regions of the gene. Predicted tertiary structure is useful for designing site-directed mutagenesis experiments.

Other types of bioinformatics tools not included in this article but useful for cancer research would be transcription binding site prediction tools (or DNA motif finding algorithms). For DNA motif finding tools, please refer to recent studies on the benchmarking of several programs (Tompa et al 2005; Hu et al 2005). All of the introduced resources can be used on-line from their websites, but some are also downloadable for use on local machines. The resources for which local copies are available are explicitly mentioned in the text because they can be integrated into a microarray data management system to make the system more comprehensive. It is no doubt that bioinformatics are going to play a more important role in cancer research in this new century, and this article is intended to be an aid for selecting useful tools for researchers in this field.

## Figures and Tables

**Table 1.** Microarray Analysis Software Focusing on Function Clustering

Software	Location	Website
GoMiner	NCI, NIH	http://discover.nci.nih.gov/gominer/
GoSurfer	Harvard Univ.	http://www.biostat.harvard.edu/complab/gosurfer/
GenMAPP	UC San Francisco	http://www.genmapp.org
ArrayTrack	US Food and Drug Administration (FDA)	http://www.fda.gov/nctr/science/centers/toxicoinformatics/ArrayTrack/index.htm

**Table 2.** Microarray Analysis Software Using Statistical Methodologies

Software/Link	Location	Website
caGEDA	Univ. of Pittsburgh	http://bioinformatics.upmc.edu/GE2/GEDA.html
SAM	Stanford Univ.	http://www-stat.stanford.edu/~tibs/SAM/
NUDGE	Univ. of Washington	http://www.bioconductor.org/
Microarray Software Comparison	The Chinese Univ. of Hong Kong	http://ihome.cuhk.edu.hk/~b400559/arraysoftrpackages.html

**Table 3.** Microarray Data Repositories

Repository	Location	Web Site
GEO	NCBI, NIH	http://www.ncbi.nlm.nih.gov/projects/geo/
ArrayExpress	EMBL-EBI	http://www.ebi.ac.uk/arrayexpress/
CIBEX	Nat. Inst. Genetics, Japan	http://cibex.nig.ac.jp
Standard Microarray Database (SMD)	Stanford Univ.	http://smd.stanford.edu/
The Gene Expression Database (GXD)	The Jackson Lab.	http://www.informatics.jax.org/mgihome/GXD/aboutGXD.shtml
Oncomine	Univ. of Michigan	http://www.oncomine.org

**Table 4.** Protein Function Prediction Tools

Software	Type	Location	Web Site
BLAST	Homology search	NCBI, NIH	http://www.ncbi.nlm.nih.gov/BLAST select protein-protein BLAST
FASTA	Homology search	Virginia Univ. Kyoto Univ.	http://fasta.bioch.virginia.edu http://fasta.genome.jp/
PSI-BLAST	Homology search	NCBI, NIH	http://www.ncbi.nlm.nih.gov/BLAST select “PSI- and PHI-BLAST”
Pfam	Protein family identification	Washington Univ	http://pfam.wustl.edu
SMART	Conserved Motif search	EMBL	http://smart.embl-heidelberg.de
PROSITE	Functional Motif search	Swiss Inst. Bioinformatics	http://us.expasy.org/prosite http://motif.genome.ad.jp
ELM	Functional motif search in eukaryotes	The ELM Consortium	http://elm.eu.org
STRING	Function prediction by comparative genomics	EMBL	http://string.embl.de
PSORT	Subcellular localization prediction	Human Genome Center, Tokyo Univ.	http://www.psort.org
PFP	Function prediction by mining PSI-BLAST result	Purdue Univ.	http://dragon.bio.purdue.edu/pfp

**Table 5.** Protein Structure Prediction Tools

Software	Type	Location	Web Site
PSIPRED	2ndary structure	Univ. College London	http://bioinf.cs.ucl.ac.uk/psipred/
PORTER	2ndary structure	Univ. College Dublin	http://distill.ucd.ie/porter/
SAM-T02	2ndary structure	UC Santa Cruz	http://www.cse.ucsc.edu/research/compbio/HMM-apps/T02-query.html
SABLE	2ndary str., solvent accesibility	Cincinnati Children’s Hospital Med. Center	http://sable.cchmc.org/
PredictProtein	2ndary structure and others	Columbia Univ.	http://cubic.bioc.columbia.edu/predictprotein/
COILS	Coiled-coil region	EMBnet, Switzerland	http://www.ch.embnet.org/software/COILSform.html
GlobPlot	Disordered region	EMBL	http://globplot.embl.de/
PONDR	Disordered region	Indiana Univ.	http://www.pondr.com/
TMHMM	Transmembrane domain	Technical Univ. of Denmark	http://www.cbs.dtu.dk/services/TMHMM-2.0/
HMMTOP	Transmembrane domain	Hungarian Academy of Sciences	http://www.enzim.hu/hmmtop/
SWISS-MODEL	3D structure; (Homology modeling)	Swiss Inst. of Bioinformatics	http://swissmodel.expasy.org
HHPred	3D str.; (Homology modeling)	Max-Planck Inst.	http://protevo.eb.tuebingenmpg.de/toolkit/index.php?view=hhpred
MODELLER	3D str.; (Homology modeling)	UC San Francisco	http://salilab.org/modeller/
FUGUE	3D str., threading	Univ. of Cambridge	http://www-cryst.bioc.camac.uk/~fugue/
Phyre	3D str., threading	Imperial College London	http://www.sbg.bio.ic.ac.uk/~phyre/
SPARKS	3D str., threading	SUNY Buffalo	http://phyyz4.med.buffalo.edu/hzhou/anonymous-fold-sparks2.html
Robetta	3D str; ab initio	Univ. Washington	http://robetta.bakerlab.org/

**Table 6.** Protein Protein Interaction Databases and Database Tools

DB/Software	Type	Location	Web Site
BIND	PPI, pathway	Mt. Sinai Hospital, Canada	http://bind.ca/
DIP	PPI	UC Los Angeles	http://dip.doe-mbi.ucla.edu/
MIPS	Mammalian PPIs	Munich Information Center for Protein Sequences	http://mips.gsf.de/proj/ppi/
HPRD	Human protein references	Johns Hopkins Univ.	http://www.hprd.org/
GRID	genetic and physical interactions of yeast, fry, worm	Mt. Sinai Hospital, Canada	http://biodata.mshri.on.ca/grid/
IntAct	open source db systems & tools for PPI data	EBI	http://www.ebi.ac.uk/intact/
Ospray	PPI visualization tool	Mt. Sinai Hospital, Canada	http://biodata.mshri.on.ca/osprey/
